# Development and Evaluation of a Prediction Model for Underestimated Invasive Breast Cancer in Women with Ductal Carcinoma In Situ at Stereotactic Large Core Needle Biopsy

**DOI:** 10.1371/journal.pone.0077826

**Published:** 2013-10-11

**Authors:** Suzanne C. E. Diepstraten, Stephanie M. W. Y. van de Ven, Ruud M. Pijnappel, Petra H. M. Peeters, Maurice A. A. J. van den Bosch, Helena M. Verkooijen, Sjoerd G. Elias

**Affiliations:** 1 Department of Radiology, University Medical Center Utrecht, Utrecht, The Netherlands; 2 Department of Radiology, Stanford University Medical Center, Stanford, California, United States of America; 3 Julius Center for Health Sciences and Primary Care, University Medical Center Utrecht, Utrecht, The Netherlands; H. Lee Moffitt Cancer Center & Research Institute, United States of America

## Abstract

**Background:**

We aimed to develop a multivariable model for prediction of underestimated invasiveness in women with ductal carcinoma in situ at stereotactic large core needle biopsy, that can be used to select patients for sentinel node biopsy at primary surgery.

**Methods:**

From the literature, we selected potential preoperative predictors of underestimated invasive breast cancer. Data of patients with nonpalpable breast lesions who were diagnosed with ductal carcinoma in situ at stereotactic large core needle biopsy, drawn from the prospective COBRA (Core Biopsy after RAdiological localization) and COBRA2000 cohort studies, were used to fit the multivariable model and assess its overall performance, discrimination, and calibration.

**Results:**

348 women with large core needle biopsy-proven ductal carcinoma in situ were available for analysis. In 100 (28.7%) patients invasive carcinoma was found at subsequent surgery. Nine predictors were included in the model. In the multivariable analysis, the predictors with the strongest association were lesion size (OR 1.12 per cm, 95% CI 0.98-1.28), number of cores retrieved at biopsy (OR per core 0.87, 95% CI 0.75-1.01), presence of lobular cancerization (OR 5.29, 95% CI 1.25-26.77), and microinvasion (OR 3.75, 95% CI 1.42-9.87). The overall performance of the multivariable model was poor with an explained variation of 9% (Nagelkerke’s *R* ^*2*^), mediocre discrimination with area under the receiver operating characteristic curve of 0.66 (95% confidence interval 0.58-0.73), and fairly good calibration.

**Conclusion:**

The evaluation of our multivariable prediction model in a large, clinically representative study population proves that routine clinical and pathological variables are not suitable to select patients with large core needle biopsy-proven ductal carcinoma in situ for sentinel node biopsy during primary surgery.

## Introduction

Since the implementation of breast cancer screening, the number of women diagnosed with ductal carcinoma in situ (DCIS) has increased [1]. DCIS is usually diagnosed preoperatively by either stereotactic or ultrasound-guided large core needle biopsy (LCNB). In approximately 26% of patients with DCIS diagnosed at LCNB, subsequent surgery reveals presence of invasive cancer (so called “DCIS underestimation”) [2]. The surgical management of patients with DCIS differs from that of patients with invasive carcinoma as the latter group requires axillary staging. Current international guidelines do not recommend axillary staging for patients diagnosed with DCIS at biopsy, except when mastectomy is planned or in the case of a large (> 5 cm) lesion with proven or suspected microinvasion [3]. Thus, in the majority of cases, underestimation of invasiveness in patients diagnosed with DCIS at LCNB will lead to an extra surgical procedure. Also, patients may potentially suffer adverse psychological effects when they are confronted with an upgrade of disease severity after surgery.

A prediction model that enables accurate identification of patients with DCIS underestimation before surgery would reduce the number of patients that need to undergo a second-step sentinel node biopsy (SNB) procedure. Many studies have reported on possible preoperative predictors of DCIS underestimation [2,4–39], but only two studies used their results to develop a multivariable prediction model and evaluated its performance. In 2011, Houssami et al. [40] developed and evaluated a multivariable model specifically for patients with microcalcifications who were diagnosed by means of vacuum-assisted biopsy. The model performance was acceptable but not generalizable to patients who underwent LCNB or had a breast lesion that did not present as microcalcifications. More recently, Park et al. [41] reported on development and validation of a multivariable model containing sonography-related variables, biopsy technique and suspicious microinvasion as predictor variables. Although they tested the model performance in a study population consisting of both women with palpable and women with nonpalpable disease and reported a reasonable model performance, presence of suspicious microinvasion is the only predictor in their model that discriminates within the group of patients with a nonpalpable lesion who are diagnosed by stereotactic LCNB. 

We aim to develop and evaluate a multivariable model build with routine clinicopathological variables to predict DCIS underestimation in women with nonpalpable breast lesions who are diagnosed through stereotactic LCNB. To do this, we use pooled data of two large prospective multicenter studies. 

## Methods

### Ethics Statement

For this report we made use of two well-established studies: the COBRA [42] (Core Biopsy after RAdiological localisation) and COBRA2000 [43] studies. Both studies were carried out in compliance with the Helsinki Declaration, and the local Institutional Review Boards of all participating institutions approved the study protocols. In accordance with the study protocols, verbal informed consent was obtained from each participant, as verbal informed consent was customary in the Netherlands at that time. 

### Patients

The COBRA study (1997-2000) was designed to investigate the diagnostic accuracy of stereotactic LCNB in women with non-palpable breast lesions. From 19 Dutch hospitals, 928 women with a non-palpable breast lesion requiring histological sampling were recruited and referred to one of five centers that specialized in stereotactic LCNB (University Medical Center Utrecht, Bosch Medical Center Den Bosch, Martini Hospital Groningen, Dr Daniel den Hoed Clinic Rotterdam or Antoni van Leeuwenhoek Hospital Amsterdam). The COBRA2000 study (2000-2003) evaluated the clinical implementation of the guidelines that were developed based on the COBRA study results. For this study, 874 women with non-palpable breast lesions scheduled for histological sampling were recruited from 40 Dutch hospitals and stereotactic LCNB was performed in one of four centers (same as COBRA, excluding Rotterdam). DCIS diagnosis on LCNB warranted subsequent surgery at the referring hospital. Both studies were approved by all institutional medical ethics committees. Together, the COBRA and COBRA2000 studies comprise 1700 consecutive patients that were sampled successfully, of whom 386 (23%) had a DCIS diagnosis at LCNB [42,43]. The majority of these patients was referred by the Dutch population-based breast cancer screening program, which consists of a two-yearly mammographic screening starting at the age of 50 years.

### Stereotactic large core needle biopsy procedure and histopathological evaluation of cores

In COBRA and COBRA2000, stereotactic LCNB was performed adhering to a standardized protocol [42]. Shortly, women were positioned prone on a biopsy table (Fisher Imaging, Denver, CO, or LORAD Stereoguide, Danbury, CT) and biopsies were taken with a 14-gauge (G), 2.2 cm excursion long throw, automated biopsy device (Biopsy gun, C.R. Bard, Covington, GA). Contraindications were: coagulopathies, use of anticoagulants, or the inability to maintain a prone position for at least one hour. The protocol included a recommendation to take at least five cores per lesion. In COBRA 2000, a minimum of eight cores was recommended when the lesion consisted of microcalcifications. Specimen mammography ascertained sampling of microcalcifications. Pathologists affiliated to the referring hospitals evaluated the LCNB specimens according to routine clinical practice [42,43].

### Data collection

In both the COBRA and COBRA2000 study, data were collected prospectively. Participants reported information on demography, medical history, breast cancer risk factors and referring route at study entry. Findings on clinical examination, mammography, and ultrasonography (if performed) were assessed preoperatively according to routine clinical practice. Some imaging and pathological variables that were essential for this study had not been (fully) documented for the COBRA and COBRA 2000 study: maximum lesion diameter, lobular cancerization, presence of necrosis and presence of microinvasion (defined as the extension of cancer cells beyond the basement membrane into the adjacent tissues with no focus more than 0.1 cm in greatest dimension). These data were retrieved from the source material at the start of the current study by observers who were blinded for the outcome. Lesion diameter was measured on the mammogram in two directions to assess the maximum lesion diameter. Data on pathology results were obtained by reviewing the pathology records. To accurately assess the presence of DCIS underestimation, the cohort was linked to the Dutch National Pathology Database (Pathologisch Anatomisch Landelijk Geautomatiseerd Archief, PALGA; December 1, 2005), enabling complete follow-up until the last surgical procedure for the index lesion. Also, the number of surgical procedures and the time between LCNB to definitive diagnosis were documented.

### Identification of preoperative predictors of DCIS underestimation

From the literature we specified a set of nine potential preoperative predictors of DCIS underestimation: age at biopsy [34,38], lesion size on diagnostic mammography [2,4,7,8,10,13–15,18,20,26,27,31,32,34,38,39], presence of a mass/density on diagnostic mammography [2,4,8,11,15–18,20,31,36], BI-RADS classification [2,31], number of cores [8,16,20,22], DCIS histological grade [2,5,6,13,18,26,28,36,38], presence of necrosis [30,32,33,38,39], lobular cancerization [12,15] and microinvasion [6,11,28]. The number of selected predictor variables did not exceed the recommended maximum of 1 predictor variable per 10 outcome events [44]. 

### Statistical analyses

First, the dataset was inspected for missing values and patterns of missingness. Missing values for preoperative predictors were imputed by multiple imputation (iterative Markov Chain Monte Carlo method) [45,46]. We used 22 variables from patient characteristics, radiological findings and core histopathology (including the outcome variable) to build the imputation model, and used this model to create 10 imputed datasets. Descriptive statistics included proportions for categorical variables. Continuous variables were analyzed according to their mean and standard deviation or median and first - third quartile. The univariable relation of each predictor with the outcome, i.e. presence/absence of DCIS underestimation was assessed using the Pearson’s Chi-square test for categorical variables and the Student’s T-test or the Mann-Whitney U test for continuous variables. Then, Binary logistic regression models were fitted, both univariable and multivariable, with DCIS underestimation as the dependent variable. Continuous variables were modeled as such and were tested for linearity by the Hosmer-Lemeshow goodness-of-fit test and also by adding quadratic transformations to the univariable models. The linearity assumption was not violated. Ordinal predictors were dichotomized. Odds ratios (OR) were reported for each predictor together with their 95% confidence intervals and the Wald test result for statistical significance. Finally, the performance of the prediction model was evaluated. We assessed overall performance of the model using Nagelkerke’s *R* ^*2*^, which indicates the percentage of variance in the outcome variable that is explained by the model. Model discrimination was assessed by the area under the curve (AUC) of the receiver operating curve and the discrimination slope. The AUC represents the probability that a patient with the outcome is given a higher probability of the outcome by the model than a randomly chosen patient without the outcome. The discrimination slope is the absolute difference in the mean predicted probabilities for the group of patients with the outcome and the group of patients without the outcome, which shows how well the two groups are separated. Calibration was tested with the Hosmer-Lemeshow goodness-of-fit test [47]. All analyses were performed with IBM SPSS Statistics, Version 20.0.0 (IBM Corp., Armonk, NY). Statistical tests were two-sided with a 5% cut-off for statistical significance. Where possible, 95% confidence intervals were reported. Results that were obtained from the multiple imputed datasets were pooled according to Rubin’s rules [48]. 

## Results

Of the 386 eligible women diagnosed with DCIS by LCNB, 26 could not be linked to PALGA. We excluded 12 more women because final diagnosis could not be ascertained (no subsequent surgery following non-representative open-breast biopsy (n=5), patients refraining from surgery after LCNB diagnosis (n=5), neoadjuvant therapy for contralateral synchronous breast cancer (n=1), ipsilateral synchronous invasive cancer (n=1)), leaving 348 (90%) women available for analysis. 

Missing value inspection showed 194 women (56%) with complete data for all variables, 116 women (33%) had one, 33 (9%) had two, and five (1%) had three or four variables missing (Table S1). All further results are derived from the data after missing value imputation. 

Mean age of the patients was 58 years (range 30 - 86). Median lesion size was 1.8 cm. The number of cores retrieved varied from 1 to 18 with a median number of 6 cores taken (Table 1). Reviewing all pathology reports through final surgery identified 100 of 348 women with DCIS underestimation at LCNB, yielding an overall DCIS underestimate rate of 28.7%, and comparable DCIS underestimate rates for the COBRA and COBRA2000 populations separately (28.5% and 28.9% respectively). Definitive treatment was achieved after 1.4 surgical procedures on average (range 1-4), and within a median time interval of 36 days (1^st^ - 3^rd^ quartile: 21 - 58 days).

**Table 1:** Patient characteristics.

		All	COBRA	COBRA2000
		(n=348)	(n=144)	(n=204)
Age in years, mean (± SD)		58.2 (10.3)	59.0 (9.9)	57.6 (10.5)
Mammographic lesion size in cm, median (1^st^ - 3^rd^ quartile)		1.8 (1.0 - 3.1)	1.3 (1.0 - 2.0)	2.1 (1.2 - 3.9)
Presence of mass or density on mammogram		66 (19 %)	40 (28 %)	26 (13 %)
Presence of microcalcifications on mammogram		328 (94 %)	132 (92 %)	196 (96 %)
Radiological classification				
	Probably benign	43 (12 %)	15 (10 %)	28 (14 %)
	Suggestive of malignancy	268 (77 %)	111 (77 %)	157 (77 %)
	Radiologically malignant	38 (11 %)	19 (13 %)	19 (9 %)
Number of core biopsies, median (1^st^ - 3^rd^ quartile)		6 (6 - 8)	7 (6 - 8)	6 (6 - 8)
DCIS^a^ histological grade				
	Low	53 (15 %)	31 (22 %)	22 (11 %)
	Intermediate	121 (35 %)	44 (31 %)	77 (38 %)
	High	174 (50 %)	69 (48 %)	105 (51 %)
Presence of necrosis in LCNB^b^-specimen		47 (14 %)	17 (12 %)	30 (15 %)
Presence of lobular cancerization in LCNB-specimen		8 (2 %)	2 (1 %)	6 (3 %)
Presence of microinvasion in LCNB-specimen		19 (5 %)	4 (3 %)	15 (7 %)

^a^ DCIS=ductal carcinoma in situ, ^b^ LCNB=large core needle biopsy

Age at biopsy and the presence of a mass/density or microcalcifications did not appear to be related to DCIS underestimation (Table 2). The risk of DCIS underestimation increased with the radiologist’s suspicion of malignancy (from 26% for probably benign (BI-RADS 3) to 38% for radiologically malignant (BI-RADS 5)), with increasing histological grade (from 27% for low to 32% for high grade), and if the biopsy showed necrosis (no necrosis: 28%; necrosis: 36%), although these relations were not statistically significant. Three predictors were univariably statistically significant: lesion size (median lesion size of 1.5 (95% C.I. (1.0 - 3.0 cm) cm in cases without DCIS underestimation vs. median lesion size of 2.1 cm (95% C.I. 1.4 - 3.8 cm) in cases with DCIS underestimation), lobular cancerization (not present: 28% risk; present: 63%), and microinvasion (not present: 27%; present 58%). 

**Table 2:** Association between DCIS^a^ underestimation and preoperative predictors.

		DCIS underestimation		
		No	Yes	*p*
Age in years, mean (± SD)		58.4 (10.4)	57.7 (10.1)	0.52^*^
Mammographic lesion size in cm, median (1^st^ - 3^rd^ quartile)		1.5 (1.0 - 3.0)	2.1 (1.4 - 3.8)	0.00^**^
Presence of mass or density on mammogram				
	No	202 (72%)	80 (28%)	
	Yes	46 (70%)	20 (30%)	0.77^***^
Radiological classification				
	Probably benign	32 (74%)	11 (26%)	
	Suggestive of malignancy	193 (72%)	75 (28%)	
	Radiologically malignant	23 (62%)	14 (38%)	0.41^***^
Number of core biopsies, median (1^st^ - 3^rd^ quartile)		7 (6 - 8)	6 (6 - 8)	0.06^**^
DCIS^a^ histological grade				
	Low	38 (73%)	14 (27%)	
	Intermediate	91 (75%)	31 (25%)	
	High	119 (68%)	55 (32%)	0.44^***^
Presence of necrosis in LCNB^b^-specimen				
	No	218 (72%)	83 (28%)	
	Yes	30 (64%)	17 (36%)	0.23^***^
Presence of lobular cancerization in LCNB-specimen				
	No	245 (72%)	95 (28%)	
	Yes	3 (38%)	5 (63%)	0.03^***^
Presence of microinvasion in LCNB-specimen				
	No	240 (73%)	89 (27%)	
	Yes	8 (42%)	11(58%)	0.00^***^

^a^ DCIS=ductal carcinoma in situ, ^b^ LCNB=large core needle biopsy

^*^ Student’s T-test, ^**^ Mann-Whitney U-test, ^***^ Pearson Chi-square test

Full mutual adjustment of the predictors did not change the associations with DCIS underestimation substantially (Table 3). In the multivariable model, the predictors with the strongest association were lobular cancerization (OR 5.29, 95% CI 1.25-26.77), microinvasion (OR 3.75, 95% CI 1.42-9.87), the number of core biopsies retrieved (OR per core 0.87, 95% CI 0.75-1.01), and lesion size (OR 1.12, 95% CI 0.98-1.28). 

**Table 3:** Logistic regression analysis of preoperative predictors of DCIS^a^ underestimation.

			Univariable		Multivariable	
			OR^b^ (95% CI^c^)	*p* ^*^	OR (95% CI)	*p* ^*^
Age in years (continuous, per year)			0.99 (0.97-1.02)	0.52	1.00 (0.97-1.02)	0.83
Mammographic lesion size in cm (continuous, per cm)			1.11 (0.98-1.25);	0.09	1.12 (0.98-1.28)	0.09
Presence of mass or density on mammogram						
	No		1.00		1.00	
	Yes		1.09 (0.60-1.98)	0.77	1.09 (0.57-2.08)	0.99
Radiological classification						
	Probably benign or suggestive of malignancy		1.00		1.00	
	Radiologically malignant		1.60 (0.77-3.33)	0.21	1.79 (0.81-3.99)	0.15
Number of core biopsies (continuous, per core)			0.92 (0.81-1.05)	0.21	0.87 (0.75-1.01)	0.07
DCIS^a^ histological grade						
		Low or intermediate	1.00		1.00	
		High	1.33 (0.78-2.27)	0.29	1.20 (0.67-2.14)	0.54
Presence of necrosis in LCNB^d^-specimen						
		No	1.00		1.00	
		Yes	1.49 (0.78-2.84)	0.23	1.50 (0.74-3.06)	0.27
Presence of lobular cancerization in LCNB-specimen						
		No	1.00		1.00	
		Yes	4.30 (1.01-18.34)	0.049	5.79 (1.25-26.77)	0.03
Presence of microinvasion in LCNB-specimen						
		No	1.00		1.00	
		Yes	3.71 (1.45-9.52)	0.006	3.75 (1.42-9.87)	0.008

^a^ DCIS=ductal carcinoma in situ, ^b^ OR=odds ratio, ^c^ CI=confidence interval, ^d^ LCNB=large core needle biopsy

* Wald test

Overall performance of the model was poor with an explained variation (Nagelkerke’s *R* ^*2*^) of 9%. Discriminative ability was mediocre with an AUC of 0.66 (95% CI: 0.58-0.73). The mean predicted probability was 0.34 in women with DCIS underestimation and 0.27 in women without DCIS underestimation, resulting in an absolute difference of 0.07 (discrimination slope). The model-derived predicted probabilities ranged from 0.06 to 0.76, with 29% of patients exceeding a predicted probability of 50%. We found no evidence for gross calibration violations (*p*-value Hosmer-Lemeshow goodness-of-fit test was non-significant).

## Discussion

In this study we developed and evaluated a multivariable model to predict underestimated invasive cancer in women with DCIS diagnosed at stereotactic LCNB for nonpalpable breast lesions. We used routinely available patient-related and procedure-related characteristics, and radiological and histopathological findings, that have previously been associated with DCIS underestimates in the literature. Nine predictors were included in the multivariable model: age at biopsy, lesion size on diagnostic mammography, presence of a mass/density on diagnostic mammography, radiological (BI-RADS) classification, number of cores, DCIS histological grade, presence of necrosis, lobular cancerization, and microinvasion. The overall performance of the multivariable model however was poor, and the ability of the model to discriminate was mediocre (AUC 0.66). Consequently, predicting DCIS underestimation using routinely available clinical or pathological information is not suitable for decision making in the clinical setting. Therefore, we refrained from proposing a clinical decision rule nor did we attempt to compensate for over-optimism of our model or model reduction.

This study has several strong points. First of all, the large number of patients (n=348), and hence events (n=100), enabled us to robustly evaluate a substantial set of predictors that we preselected from the literature. Secondly, the participation of 40 different hospitals in the Netherlands ensures a good representation of routine clinical practice, which indicates that our results reflect the true clinical applicability of the model. Thirdly, thorough follow-up of patients for final diagnosis minimized the possibility of misclassification with respect to the outcome. Most of the previously published studies did not report follow up of patients after the first surgery, except for Leikola et al. [21]. Since approximately 40% of women with DCIS need more than one surgery before final treatment is achieved [49], the extended follow-up time is a major strength in our study. Finally, we used multiple imputation to impute the missing data in our dataset, which decreases the chance of selection bias and provides the most reliable estimates as compared to complete case analysis or single imputation techniques [45,46]. However, in a substantial proportion of cases (44%) one or more values were missing, and although multiple imputation provides highly reliable estimates, prevention of missing values in a dataset is always best.

A limitation of the current study is that the generalizability is restricted to women with a nonpalpable breast lesion undergoing stereotactic 14G LCNB. Today, many breast clinics use vacuum-assisted biopsy as their method of choice to sample nonpalpable breast lesions, since this method has been proven to substantially reduce the number of DCIS underestimates [2,50]. Nonetheless, LCNB is still used, and no multivariable model to predict DCIS underestimation for this population has been described as of yet.

Two research groups have previously reported on the performance of a multivariable model for predicting DCIS underestimation. In 2011 Houssami et al. [40] tested the discriminative ability of several models containing preoperative variables to predict DCIS underestimation in women with microcalcifications who were diagnosed with DCIS on vacuum-assisted biopsy. They tested models that included age, lesion size, distribution and morphology of microcalcifications on the mammogram, radiological classification, number of cores, and DCIS histological grade with a stepwise forward approach, and reported AUCs for every step varying from 0.70 for a model with lesion size only to 0.76 for a full model containing all predictors. They found that DCIS histological grade was the only predictor that significantly improved the model fit beyond lesion size. A unique aspect of their study was that they compared the measurements of two observers for the imaging variables, and evaluated a prediction model for each observer separately. Since interobserver variability will likely decrease the predictive power of a predictor variable, this may be the reason that their prediction models yielded larger AUCs than ours. More recently, Park et al. [41] developed a prediction model for DCIS underestimation using retrospectively collected data from 340 patients that were diagnosed with DCIS by either 14 G LCNB or vacuum-assisted biopsy in a single institution. The predictor variables in the model were derived from their own dataset and included palpability of the lesion, presence of calcifications on ultrasound, presence of a mass on ultrasound, biopsy method (CNB vs. vacuum-assisted biopsy), and presence of suspicious microinvasion. Their model performance was fairly good with an AUC of 0.76, which was reduced to 0.71 after internal validation. However, their model would function as a univariable prediction model with presence of suspicious microinvasion as the predictor variable for our study population because it consists of patients with nonpalpable lesions that are sampled with stereotactic 14G LNCB. Since this predictor is also included in our model, it’s clear that the proposed model would perform less well in our study population. Another reason why the model would perform differently in our population is that in contrast to Park et al. we do not consider microinvasion at final surgery to be a DCIS underestimate.

The observed associations between predictor variables and DCIS underestimation in our dataset largely agreed with what is reported in the literature. In the multivariable model, the predictors mammographic lesion size, radiological classification, DCIS histological grade, necrosis, lobular cancerization, and microinvasion were found to have a positive association with DCIS underestimation. Number of cores retrieved at biopsy was negatively associated with underestimated invasive. 

It was noted that necrosis was reported markedly less in our population compared to other studies, i.e. 14% in our study compared to 33-77% in published studies [8,10,12,15,17–21,24,25,27,29–33,38], although Goyal et al reported a frequency as low as 3% [4]. Also, lobular cancerization was less prevalent in our population (2%) compared to other studies (14-64%) [8,12,15,29,33]. In our large cohort the histopathology assessments were not made by experts, but rather by regular pathologists. This could be one of the reasons for the underreporting of presence of necrosis and lobular cancerization. If pathologists in a certain hospital never routinely report presence of lobular cancerization or necrosis, this will lead to misclassification. Most probably in this case the misclassification will be at random, i.e. not related to the outcome (DCIS underestimation). Random misclassification will almost always lead to underestimation of results [51], meaning underestimation of the predictive value in our case. A reassessment of these particular potential predictors by an expert pathologist may thus have a positive influence on the performance of our prediction model and risk score. However, assessment by regular pathologists does make the data more representative of the routine clinical setting, which is of importance in determining the true clinical applicability of the model. 

Number of core biopsies showed a negative relation with the outcome which was corresponding to the literature [8,14,16,19,20,30], except for the studies of Trentin et al. [34] and Huo et al. [15] reporting no relation and a positive (non-significant) relation respectively. Moreover, other biopsy techniques that yield a larger tissue volume, such as vacuum assisted biopsy or percutaneous intact specimen biopsy have been shown to be associated with a lower probability of DCIS underestimation [2,52]. Obtaining a larger tissue volume, either by increasing the number of cores or choosing a larger needle size, is thus advisable to prevent underestimation of invasiveness in women with nonpalpable breast lesions. Furthermore, acquiring additional (non-routine) variables such as diffusion weighted MRI [53], gene expression data [54], FDG-PET or other molecular imaging assays may be worth considering in de development of a better prediction model.

In conclusion, our prediction model based on routine clinicopathological variables to predict DCIS underestimation provides insufficient performance to select patients for SNB after diagnosis of DCIS on LCNB. The fact that we evaluated a large cohort representative of routine clinical practice indicates that there is need for the identification of additional predictors of DCIS underestimation, which are not routinely available today.

## Supporting Information

Table S1 **Table of missing value analysis.**
(DOC)
