# Supplementary material for: Development and Evaluation of a Prediction Model for Underestimated Invasive Breast Cancer in Women with Ductal Carcinoma In Situ at Stereotactic Large Core Needle Biopsy
Source: PLoS One. 2013 Oct 11;8(10):e77826. doi: 10.1371/journal.pone.0077826 (PMC3795649; doi:10.1371/journal.pone.0077826)
Supplement: Table S1 — Table of missing value analysis. (DOC) [file pone.0077826.s001.doc]

| Table S1. Table of missing value analysis | | |
| --- | --- | --- |
|  | | *N (%)* |
| Number of missing values per predictor variable | |  |
|  | DCIS^a^ histologic grade | 77 (22) |
|  | Maximum lesion diameter on mammogram | 76 (22) |
|  | Radiological classification | 21 (6) |
|  | Number of cores | 18 (5) |
|  | Presence of mass/density on mammogram | 7 (2) |
|  | Age | 0 (0) |
|  | Presence of lobular cancerization | 0 (0) |
|  | Presence of necrosis | 0 (0) |
|  | Presence of microinvasion | 0 (0) |
| Number of cases with n missing values | |  |
|  | No variables missing | 194 (56) |
|  | One variable missing | 116 (33) |
|  | Two variables missing | 33 (5) |
|  | Three or four variables missing | 5 (1) |
| Patterns of missingness | |  |
|  | None | 194 (55.7) |
|  | DCIS histologic grade | 53 (15.2) |
|  | Maximum lesion diameter | 44 (12.6) |
|  | Maximum lesion diameter + DCIS histologic grade | 19 (5.6) |
|  | Number of cores | 12 (3.4) |
|  | Maximum lesion diameter + Radiological classification | 9 (2.6) |
|  | Radiological classification | 5 (1.4) |
|  | Presence of mass/density | 2 (0.6) |
|  | Radiological classification + DCIS histologic grade | 2 (0.6) |
|  | Maximum lesion diameter + presence of mass/density + radiological classification + number of cores | 2 (0.6) |
|  | Number of cores + DCIS histologic grade | 2 (0.6) |
|  | Presence of mass/density + maximum lesion diameter + radiological classification | 1 (0.3) |
|  | Presence of mass/density + radiological classification + number of cores | 1 (0.3) |
|  | Maximum lesion diameter + number of cores | 1 (0.3) |
|  | Presence of mass/density + radiological classification + DCIS histologic grade | 1 (0.3) |

^a^DCIS=ductal carcinoma in situ
